# Carbon Nanotubes as Fluorescent Labels for Surface Plasmon Resonance-Assisted Fluoroimmunoassay

**DOI:** 10.3390/s17112569

**Published:** 2017-11-07

**Authors:** Hiroki Ashiba, Yoko Iizumi, Toshiya Okazaki, Xiaomin Wang, Makoto Fujimaki

**Affiliations:** 1 Electronics and Photonics Research Institute, National Institute of Advanced Industrial Science and Technology (AIST), Tsukuba, Ibaraki 305-8565, Japan; m-fujimaki@aist.go.jp; 2 CNT-Application Research Center, National Institute of Advanced Industrial Science and Technology (AIST), Tsukuba, Ibaraki 305-8565, Japan; iizumi-yoko@aist.go.jp (Y.I.); toshi.okazaki@aist.go.jp (T.O.); 3 Nanoelectronics Research Institute, National Institute of Advanced Industrial Science and Technology (AIST), Tsukuba, Ibaraki 305-8565, Japan; wang_x105@optoquest.co.jp

**Keywords:** biosensor, carbon nanotube, fluorescent probe, surface plasmon resonance, virus detection

## Abstract

The photoluminescence properties of carbon nanotubes (CNTs), including the large Stokes shift and the absence of fluorescent photobleaching, can be used as a fluorescent label in biological measurements. In this study, the performance of CNTs as a fluorescent label for surface plasmon resonance (SPR)-assisted fluoroimmunoassay is evaluated. The fluorescence of (8, 3) CNTs with an excitation wavelength of 670 nm and an emission wavelength of 970 nm is observed using a sensor chip equipped with a prism-integrated microfluidic channel to excite the SPR. The minimum detectable concentration of a CNT dispersed in water using a visible camera is 0.25 μg/mL, which is equivalent to 2 × 10^10^ tubes/mL. The target analyte detection using the CNT fluorescent labels is theoretically investigated by evaluating the detectable number of CNTs in a detection volume. Assuming detection of virus particles which are bound with 100 CNT labels, the minimum number of detectable virus particles is calculated to be 900. The result indicates that CNTs are effective fluorescent labels for SPR-assisted fluoroimmunoassay.

## 1. Introduction

Carbon nanotubes (CNTs) have attracted considerable attention from researchers because of their superior electrical, mechanical, and optical properties [1,2]. In recent years, the photoluminescence of CNTs in the near-infrared region (1000–1400 nm) [3,4] has been utilized to develop fluorescent labels for biological experiments [5,6]. These CNT labels exhibit a large Stokes shift between the excitation and emission wavelengths, thus, the noises arising from the stray excitation light and autofluorescence of sensor chip substrates can be effectively removed using optical filters, and a high signal-to-noise ratio can be achieved. In addition, CNTs exhibit no fluorescent photobleaching that allows stable fluorescent measurements. However, a low quantum yield of CNTs, at most several per cent [3,7,8,9], limits the performance of fluorescent labels. 

Surface plasmon resonance-assisted fluoroimmunoassay (SPRF), or surface plasmon-enhanced fluorescence spectroscopy (SPFS), is a sensitive biosensing technique that uses a fluorescent label and surface plasmon resonance (SPR) [10,11,12,13]. In SPRF, the luminescence of fluorescent labels is enhanced by two effects: (i) enhancement of the electric field intensity of the excitation light and (ii) enhancement of the quantum yield of fluorescent labels. The quantum yield enhancement has a greater effect when the original quantum yield of a fluorescent label is lower [14]. In addition, for conventional SPR sensors [15,16], carbon nanomaterials including CNTs and graphene has been used to enhance the resonance shift signal [17,18]; the combination of CNT and SPR is considered to be promising. Therefore, the use of CNT fluorescent labels with SPRF is effective in improving the low quantum yield.

The authors reported a norovirus detection system based on SPRF using a CdSe quantum dot fluorescent label, which exhibits a large Stokes shift similar to CNTs, and demonstrated sensitive detection of norovirus virus-like particles (VLPs) [19]. Although CdSe quantum dots possess good optical properties as a fluorescent label, the disposal of quantum dots is problematic due to the presence of toxic heavy metals. This problem, in some cases, would be critical for practical use. In contrast, CNTs can be incinerated, and therefore, are more suitable for practical use. CNT fluorescent labels are thus worth investigating with SPRF.

In this study, the performance of CNTs as a fluorescent label for target analyte detection using SPRF was evaluated. A V-trench biosensor, which was developed as a miniature and simple SPRF apparatus [19,20,21], was used for the evaluation. The sensor is equipped with a V-shaped microfluidic channel, which functions as a prism to excite the SPR, and it can perform sensitive biosensing based on SPRF. SPR-induced fluorescence emission on the V-trench was confirmed for fluorescent molecules and quantum dot fluorescent labels in previous studies [19,20,21]. Herein, the evaluation of a CNT fluorescent label using a V-trench biosensor designed for CNTs is presented.

## 2. Materials and Methods 

### 2.1. CNT Dispersion Preparation

An aqueous dispersion of CNTs was prepared using single-strand deoxyribonucleic acid (DNA; Sonicated Salmon Sperm DNA, Agilent Technologies, Santa Clara, CA, USA). A 10 mg/L aqueous solution of the DNA was boiled for 5 min and cooled with ice. CNTs (CoMoCAT SG65, SouthWest NanoTechnologies, Canton, MA, USA) with a total mass of 20 mg were pre-dispersed in 10 mL of ethanol by bath sonication for 2 min. The CNTs were washed by repetitive vacuum filtering and sonic dispersion with 10 mL of ethanol 3 times and 10 mL of boiled water 16 times. The washed CNTs with a mass of 1 mg and 0.4 mL of the DNA solution were mixed with 10 mL of 50 mM phosphate buffer (pH 8.0). The mixture was sonicated using a VCX 500 instrument (Sonics & Materials, Newtown, CT, USA) for 10 min and centrifuged at 171000G for 60 min. In the present experiment, the supernatant containing monodisperse CNTs was used. 

**Figure 1:**
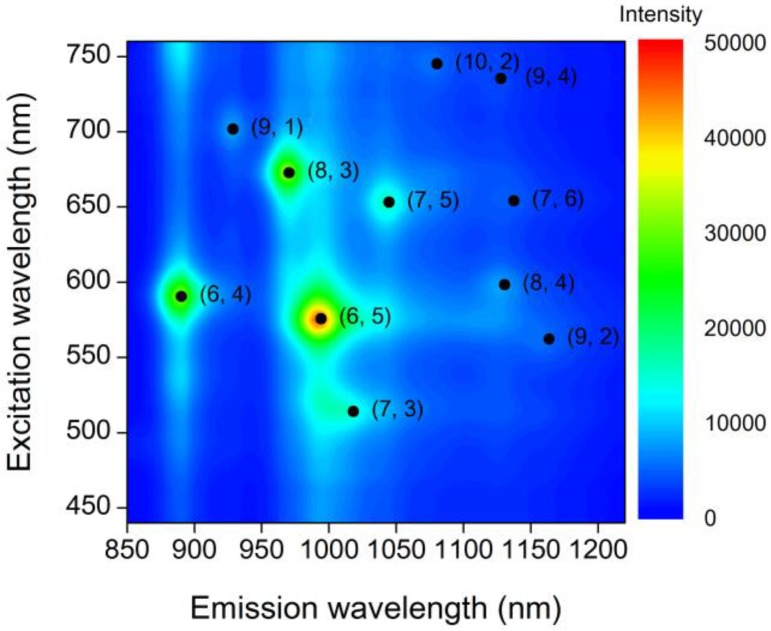
Photoluminescence map of the CNT dispersion used here.

In this study, we focused on (8, 3) CNTs with excitation and emission wavelengths of 670 nm and 970 nm, respectively. (8, 3) CNTs were chosen for the following reasons: (i) the emission at a wavelength of 970 nm is detectable using a visible charge-coupled device (CCD) camera with a relative response of several percent; (ii) the excitation at a wavelength of 670 nm is suitable for SPR excitation by gold, which is stable and is the most commonly used material for SPR. A photoluminescence map of the dispersion of CNTs measured using a spectrometer (Fluorolog-3-2-iHR320, Horiba Jobin Yvon, Kyoto, Japan) is shown in Figure 1. From the integrated photoluminescence intensity, the content ratio of the (8, 3) CNTs in the dispersion was estimated to be approximately 15%. The length of the CNTs contained in the dispersion was measured using an atomic force microscope (AFM). The AFM image is shown in Figure S1 in the Supplementary Material. The typical length of the CNT was 1 μm. The monodispersity of the CNTs was also evaluated using the AFM image, and over 90% of CNTs was monodisperse in the prepared dispersion. 

### 2.2. Experimental Apparatus

**Figure 2:**
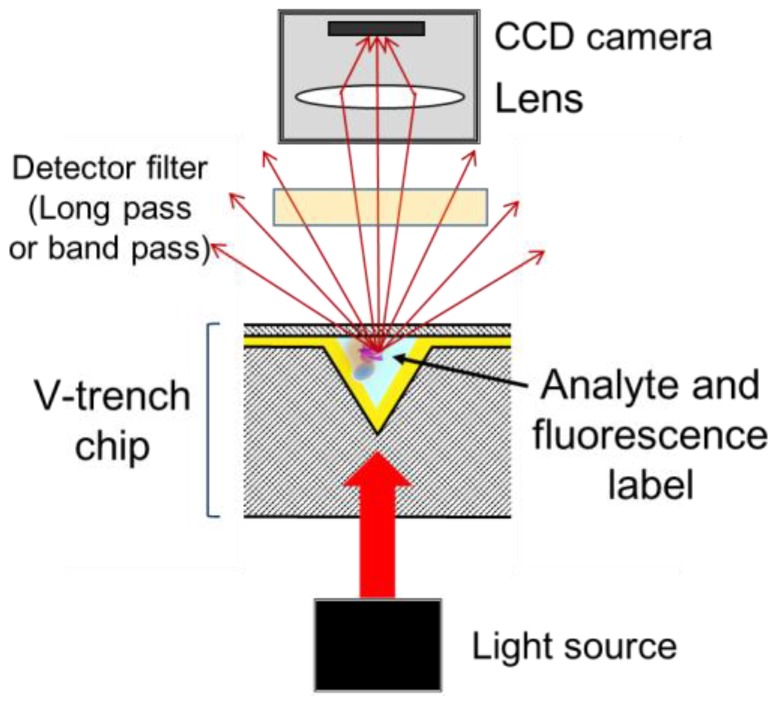
Schematic diagram of the V-trench biosensor.

A schematic diagram of the V-trench biosensor is shown in Figure 2. The optical system consists of a light source, sensor chip, and camera that are aligned in a straight line. The sensor chip is equipped with a microfluidic channel having a V-shaped cross section, which functions as a prism to excite the SPR. The vertex angle of the V-shaped trench determines the incident angle of the excitation light, and is designed using electric field simulation to maximize the electric field intensity at the surface (Section 2.3). The analyte is captured at the surface of the V-shaped trench and labeled with a fluorescent dye. The excitation light enters the sensor chip from the backside and illuminates the V-trench to excite the SPR, and the fluorescence excited by the SPR is detected using the camera. 

For the measurement of fluorescence from (8, 3) CNTs, a fluorescence imaging instrument (Light-Capture II, Atto, Tokyo, Japan) equipped with a laser diode with a wavelength of 670 nm (Shibasaki, Chichibu, Japan) is used. The excitation light, which is 6 mm in diameter, is p- or s-polarized to perform the fluorescence measurement with or without SPR excitation, respectively. The emitted light from the CNTs excited by the SPR passes through a long pass filter with a cut-off wavelength of 900 nm (FEL0900, Thorlabs, Newton, NJ, USA) and is detected using a cooled visible CCD camera (pixel size: 8.4 μm (H) × 9.8 μm (V)).

### 2.3. Sensor Chip

**Figure 3:**
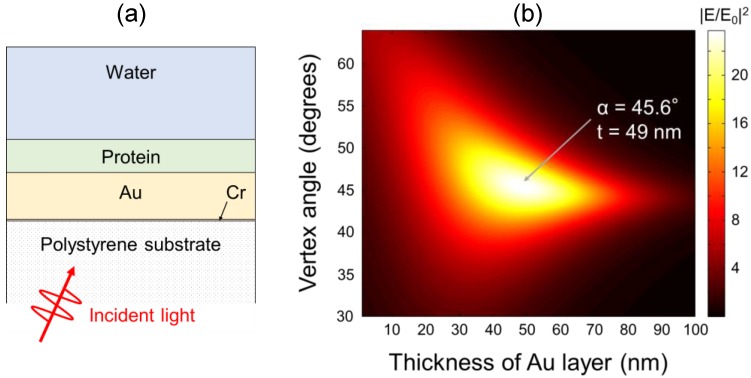
(**a**) Schematic of the cross section of the V-trench sensor chip surface used in the electric field simulation of a multi-layer model based on the transfer matrix method; (**b**) Calculated electric field enhancement factor (|E/E_0_|^2^) against the thickness of the gold layer (*t*) and the vertex angle of the V-trench (*α*). The excitation wavelength is 670 nm, and |E/E_0_|^2^ at the boundary between the protein and water layers is shown. The arrow indicates the point of maximum |E/E_0_|^2^.

The V-trench sensor chip was designed using electric field simulation based on the transfer matrix method [22]. The vertex angle of the V-trenches and the thickness of a gold film as an SPR excitation layer were determined to maximize the electric field intensity against the excitation light with a wavelength of 670 nm. A multi-layer model was used to represent the V-trench sensor chip, as shown in Figure 3a, which includes, from bottom to top, a polystyrene substrate, an adhesive layer of chromium with a thickness of 0.6 nm, a gold layer, a protein layer with a thickness of 20 nm, and water. The protein layer represents the surface modifications to the sensor chip, including immobilized antibodies and self-assembled monolayers for protein immobilization. The complex refractive indices at a wavelength of 670 nm were 1.5848 for polystyrene [23], 3.7176 + *i*4.3666 for chromium [24], 0.16316 + *i*3.4625 for gold [25], 1.45 for protein, and 1.3302 for water [26]. An electric field enhancement factor (|E/E_0_|^2^) map against the p-polarized excitation light with a wavelength of 670 nm is shown in Figure 3b, where the values of |E/E_0_|^2^ are calculated at the boundary of the protein and water layers. The maximum value of |E/E_0_|^2^ is 23.8, which is obtained at a vertex angle of 45.6° and a gold thickness of 49 nm. Thus, the sensor chip having the V-shaped trench with a vertex angle of 45.6° was prepared.

The substrate of the V-trench sensor chip was fabricated using injection molding (Cluster Technology, Higashi-Osaka, Japan) of polystyrene (CR-3500, DIC, Tokyo, Japan). The width of the opening and bottom and the length of the V-trench were 0.3, 0.02, and 10 mm, respectively. The chromium and the gold layers were formed on the substrate using a vacuum deposition system (Biemtron, Shirosato, Japan). The setting thicknesses of the deposited chromium and gold films were 0.6 nm and 126 nm, respectively. Under this condition, the thickness of the gold film in the direction perpendicular to the surface of the 45.6° V-trenches becomes 49 nm.

### 2.4. Fluorescence Measurement of CNTs

The CNT dispersion was directly applied to the V-trenches without any surface modifications. The intensities of fluorescence of the CNT dispersion samples at various concentrations were measured. 

Before applying the samples, the V-trench sensor chips were cleaned using a plasma cleaner (PDC-32G, Harrick Plasma, Ithaca, NY, USA) at an RF power of 18 W for 30 s. To acquire “Blank” fluorescent images, the sensor chip filled with 15 μL of Milli-Q water (Merck, Darmstadt, Germany) was placed in the V-trench biosensor system, the excitation light was irradiated, and the images were acquired with an exposure time of 1 min. Then, the Milli-Q water was removed from the V-trench by blowing nitrogen, and 15 μL of the CNT dispersions samples was applied. The concentration of the original CNT dispersion was 25 μg/mL, and the CNT dispersion with various concentrations ranging from 0.063 to 25 μg/mL were prepared by diluting the original dispersion with Milli-Q water. Under the excitation light irradiation, fluorescent images of the samples were acquired with an exposure time of 1 min. The luminescence intensity was obtained by integrating the brightness of each pixel in a defined area (80 × 20 pixels; approximately 7 × 2 mm^2^ on the actual dimension) of the fluorescent images. The power density of the excitation light illuminated on the sensor chip was 11 mW/cm^2^. It is known that CNTs release heat by the photothermal effect [27,28]. The effect of heat to the sensor chip and fluorescent signal was not observed for the power density used here (see Figure S2 in the Supporting Material). 

## 3. Results

**Figure 4:**
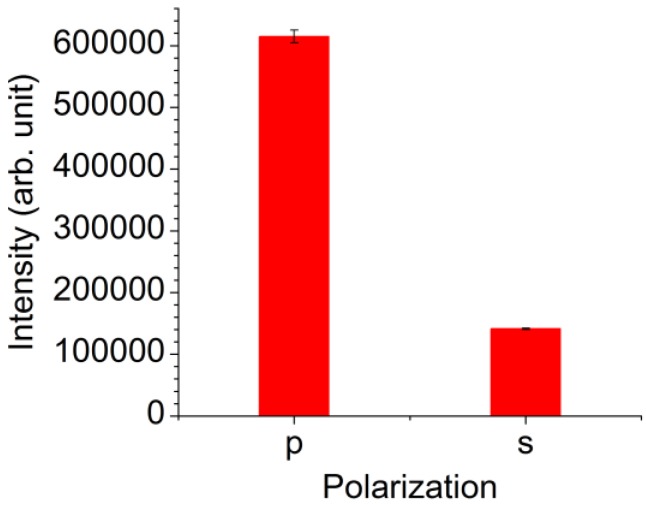
Luminescent intensities of the CNT dispersion sample with a concentration of 25 μg/mL measured using a V-trench biosensor. The excitation light was p- or s-polarized. The error bars indicate the standard errors.

The luminescent intensities of the CNT dispersion sample with a concentration of 25 μg/mL measured using the V-trench biosensor under the p- and s-polarized excitation light are shown in Figure 4. The bars in the figure indicate the average luminescent intensities obtained from three fluorescent images, and the error bars indicate the standard errors. The luminescent intensity measured under the p-polarized light irradiation, where SPR was excited, was 4.4-folds greater than that measured under the s-polarized light. This result indicates that the SPR was excited on the V-trench and that the fluorescence of CNTs was excited by the enhanced electric field of SPR. 

**Figure 5:**
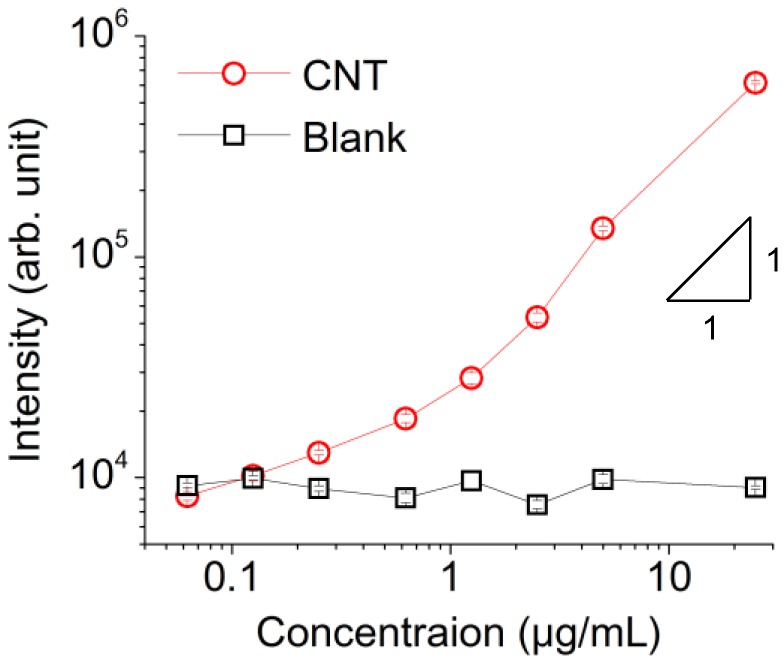
Luminescent intensities of the CNT dispersion samples with various concentrations measured using a V-trench biosensor. The excitation light was p-polarized. “CNT” and “Blank” were measured by applying the CNT dispersion samples and Milli-Q water into the V-trench, respectively. The error bars indicate the standard errors.

The luminescent intensities of the CNT dispersion samples with various concentrations measured under the p-polarized excitation light are shown in Figure 5. The symbols in the figure indicate the average luminescent intensities obtained from three fluorescent images, and the error bars indicate the standard errors. The fluorescence intensities were positively correlated with the concentration of the CNTs. By comparing the luminescent intensities of “Blank” and “CNT,” the minimum detectable concentration of the CNTs in this experiment was 0.25 μg/mL. The intensity is linear to the concentration at the range of approximately over 1 μg/mL, whereas the gradient of the intensity decreases below 1 μg/mL. This would be due to the attachment of CNTs to the sensing surface. Assuming that a density of CNTs at the sensing surface increases due to the attachment, it would result in increased luminescent intensity and the decreased gradient when the concentration is low. When the concentration is high, the attachment to the surface would be saturated and the luminescent intensities become linear.

## 4. Discussion

For evaluating the potential of CNT fluorescent label, the minimum detectable number of CNTs is calculated herein, and the minimum detectable number of target analyte, including protein molecules and virus particles, is discussed accordingly. In the fluorescent measurement described above, CNTs were considered to be uniformly distributed in the dispersion. Then, the number of CNTs contained in a detection volume, *N_CNT_*, is calculated as:

(1) $$N_{CNT}=D_{CNT}AH$$

where *D_CNT_* is the number of CNTs in a unit volume, *A* is the measurement area, and *H* is the height of a detection volume. In general, *H* is related with the height of the illumination by excitation light, since the dimension of a fluorescent label is smaller than the height of illumination. However, in this study, the CNT length is considered to be larger than the height of illumination by SPR. Specifically, the CNT length was typically 1 μm as mentioned in Section 2.1, and from the electric field simulation described in Section 2.3, the decay length of the electric field of SPR was derived to be 176 nm for the developed system. In this case, if the height of illumination is employed as *H*, the number of CNTs is underestimated. Therefore, the CNT length (1 μm) is herein employed as *H*. Next, *D_CNT_* is calculated from the concentration of CNT dispersion as follows. For example, when the concentration of dispersion is 0.25 μg/mL, the concentration of luminous (8, 3) CNTs is 3.8 × 10^−2^ μg/mL, since the content ratio of (8, 3) CNTs was approximately 15%, as mentioned in Section 2.1. The structural model of (8, 3) CNTs yielded the number of carbon atoms in its unit length as 93 atoms/nm. Using the number of carbon atoms and the CNT length (1 μm), a concentration of 3.8 × 10^−2^ μg/mL yields *D_CNT_* = 2.0 × 10^10^ tubes/mL. *A* is calculated to be 4.3 mm^2^ for the developed V-trench biosensor using the excitation light with a diameter of 6 mm and an opening width, a bottom width, and a vertex angle of the V-trench of 0.3 mm, 0.02 mm, and 45.6°, respectively. When 0.25 μg/mL is employed as the minimum detectable concentration, using Equation (1) with *D_CNT_* = 2.0 × 10^10^ tubes/mL, *A* = 4.3 mm^2^, and *H* = 1 μm, the minimum detectable number of CNTs is calculated to be 9 × 10^4^ tubes. 

As mentioned in Section 3, the luminescent intensities shown in Figure 5 reveal non-linearity at the low concentration region, which would be due to the attachment of CNTs to the sensing surface. This means that, if the attachment to the sensing surface is avoided, the minimum detectable concentration would be larger than 0.25 μg/mL. Considering the gradient of the intensities over 1 μg/mL, the minimum detectable concentration without CNT attachment is considered to be greater up to twice. Thus, the minimum detectable number of CNTs is considered to be in between 9 × 10^4^ and 2 × 10^5^ tubes.

**Figure 6:**
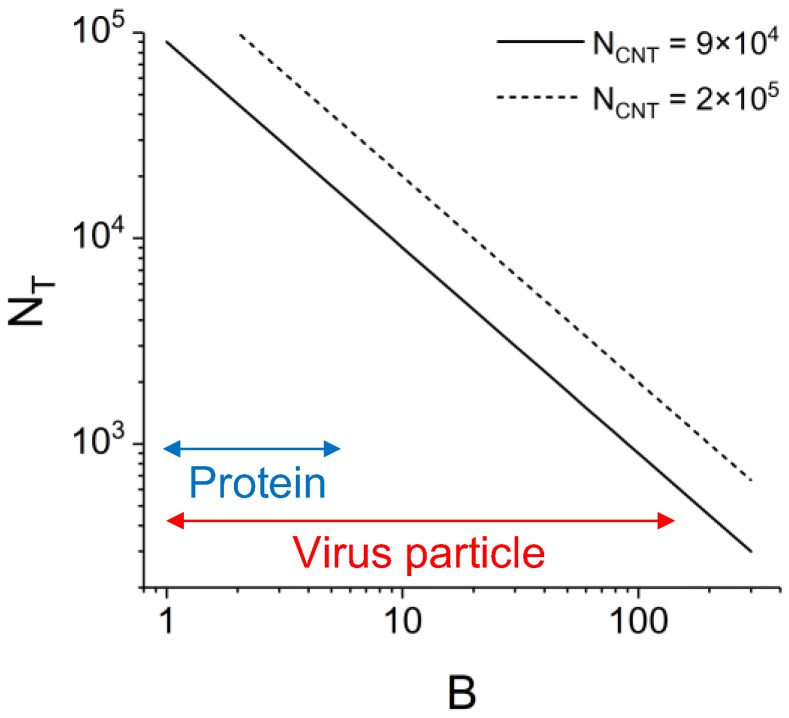
Detectable number of target analyte (*N_T_*) against the number of CNT labels bound to one target analyte (*B*) for the detectable number of CNTs (*N_CNT_*) of 9 × 10^4^ and 2 × 10^5^ tubes. Blue and red arrows indicates typical ranges of *B* for proteins and virus particles.

When considering the detection of target analyte using a CNT fluorescent label, since the CNT length is larger than the height of illumination and the dimension of target analyte, the detectable number of target analyte, *N_T_*, is simply calculated as *N_T_* = *N_CNT_*/*B*, where *B* is the number of CNT labels bound to one target analyte. The value of *B* varies depending on various factors such as the structure of target analyte, binding effectivity of a CNT label, etc. Figure 6 shows *N_T_* against *B* for *N_CNT_* = 9 × 10^4^ and 2 × 10^5^ tubes. Typical ranges of *B* for proteins and virus particles are also indicated. For example, when the CNT label is used for the detection of virus with *B* = 100, the minimum detectable number of virus particles is 900–2000. The calculation indicates that CNTs possess the potential as a fluorescent label for sensitive biosensing using SPRF sensors.

The effect of quenching in the fluorescence measurements should also be discussed. Since the surface of the V-trench is bare gold, the photoluminescence of fluorescent labels near the surface is likely quenched. Firstly, it has been reported that CNTs coated with organic molecules are less affected by quenching [29]. The CNTs used in this study were coated with DNA molecules and thus considered tolerant against quenching. Secondly, the minimum detectable concentration of CNTs was measured by including the quenching effect. That is to say, the minimum detectable number of target analyte evaluated above also includes the quenching effect. Since the CNTs are long, as mentioned above, even if a part of the CNT is inside the quenching distance, the other part of the CNT would likely be outside. Other fluorescent labels such as fluorescent molecules and quantum dots are small (<20 nm), and if these fluorescent labels come close to the surface, their luminescence intensities are significantly affected by quenching. The tolerability against quenching is an advantage of the CNT compared with other fluorescent labels.

In the fluorescence measurement, the CNT dispersions were directly applied onto the gold layer of the V-trenches, whereas, as shown in Section 2.3, the structure of the V-trench sensor chip was optimized assuming that a protein layer was formed on the gold layer to capture the target. |E/E_0_|^2^ of the V-trenches used for the CNT dispersion measurement is considered to be lower than the optimal condition; the simulation for the V-trenches without the protein layer yields |E/E_0_|^2^ = 12.6, which is half the optimal value. Therefore, in practical target detection using the V-trenches with a protein layer, the minimum detectable number of CNTs can be better than evaluated above. On the other hand, under practical conditions, various factors would depress the performance of target detection by lowering the value of *B* and generating noise. These factors includes labeling efficiency of the CNT to the antibody, binding efficiency of the antibody to the target analyte, nonspecific binding of the CNT-labeled antibody, and content ratio of luminous CNTs. In particular, labeling with antibodies is a major challenge for CNTs, because of poor reactivity. Although several techniques have been reported for CNT labeling [5,30,31], the efficiency, stability, and controllability of the labeling were insufficient. The binding of the unlabeled antibodies to the target analyte compete with that of the CNT-labeled antibodies, resulting in depressed sensitivity of target detection. Further investigation of the labeling techniques would be beneficial for CNT fluorescent labels. Regarding the content ratio of the CNTs, the CNT dispersion used in this study contained 15% of (8, 3) CNTs that emitted the observed fluorescence. It is desirable that only (8, 3) CNTs are contained in the fluorescent labels. In recent years, effective methods of selective growth and purification of CNTs have been reported [32,33]. These methods are useful to prepare efficient CNT fluorescent labels. In addition, the performance of CNT fluorescent labels can be further improved by modifying the CNT itself. For example, the luminescent intensity of CNTs is greatly enhanced by introducing defects using oxidization or covalent functionalization [34,35,36]. These techniques improve the potential of CNT labels even further. 

## 5. Conclusions

The performance of (8, 3) CNTs as a fluorescent label for SPRF was evaluated. The fluorescent measurement of CNTs was performed using a V-trench biosensor, and its capability for the influenza virus detection was estimated. A sensor chip optimized for (8, 3) CNTs was designed to enhance fluorescence with an excitation wavelength of 670 nm. The minimum detectable concentration of the CNTs was 0.25 μg/mL. The minimum detectable number of CNT labels was evaluated to be 9 × 10^4^–2 × 10^5^ tubes. The detectable number of target analyte was evaluated as the function of the minimum detectable number of CNTs and the number of CNT labels bound to one target analyte. Once the challenges such as labeling efficiency are overcome, CNTs are considered as a promising candidate for use as fluorescent labels for SPRF.

## Supplementary Materials

The following are available online at http://www.mdpi.com/1424-8220/17/11/2569/s1, Figure S1: Atomic force microscope image of the CNT dispersion. Figure S2: Luminescent intensities of the CNT dispersion sample measured with various powers of the excitation light.
