# Supplementary material for: Carbon Nanotubes as Fluorescent Labels for Surface Plasmon Resonance-Assisted Fluoroimmunoassay
Source: Sensors (Basel). 2017 Nov 7;17(11):2569. doi: 10.3390/s17112569 (PMC5713471; doi:10.3390/s17112569)
Supplement: Supplementary file 1 [file sensors-17-02569-s001.docx]

Supplementary Material

Carbon nanotubes as fluorescent labels for surface plasmon resonance-assisted fluoroimmunoassay

Hiroki Ashiba^*^, Yoko Iizumi, Toshiya Okazaki,
Xiaomin Wang, and Makoto Fujimaki

* h.ashiba@aist.go.jp


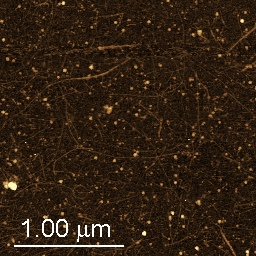


**Figure S1.** Atomic force microscope (AFM) image of the carbon nanotube (CNT) dispersion used in this study. A droplet of the CNT dispersion was put on a silicon substrate and naturally dried to immobilize CNTs. Narrow structures in the figure are CNTs. Approximately 90 CNTs are included in the figure. The typical length of the CNT is 1 μm. The height of CNTs were evaluated using the AFM image, and CNTs thinner than 1.5 nm were considered to be monodisperse. From this image, over 90% of CNTs are determined to be monodisperse.


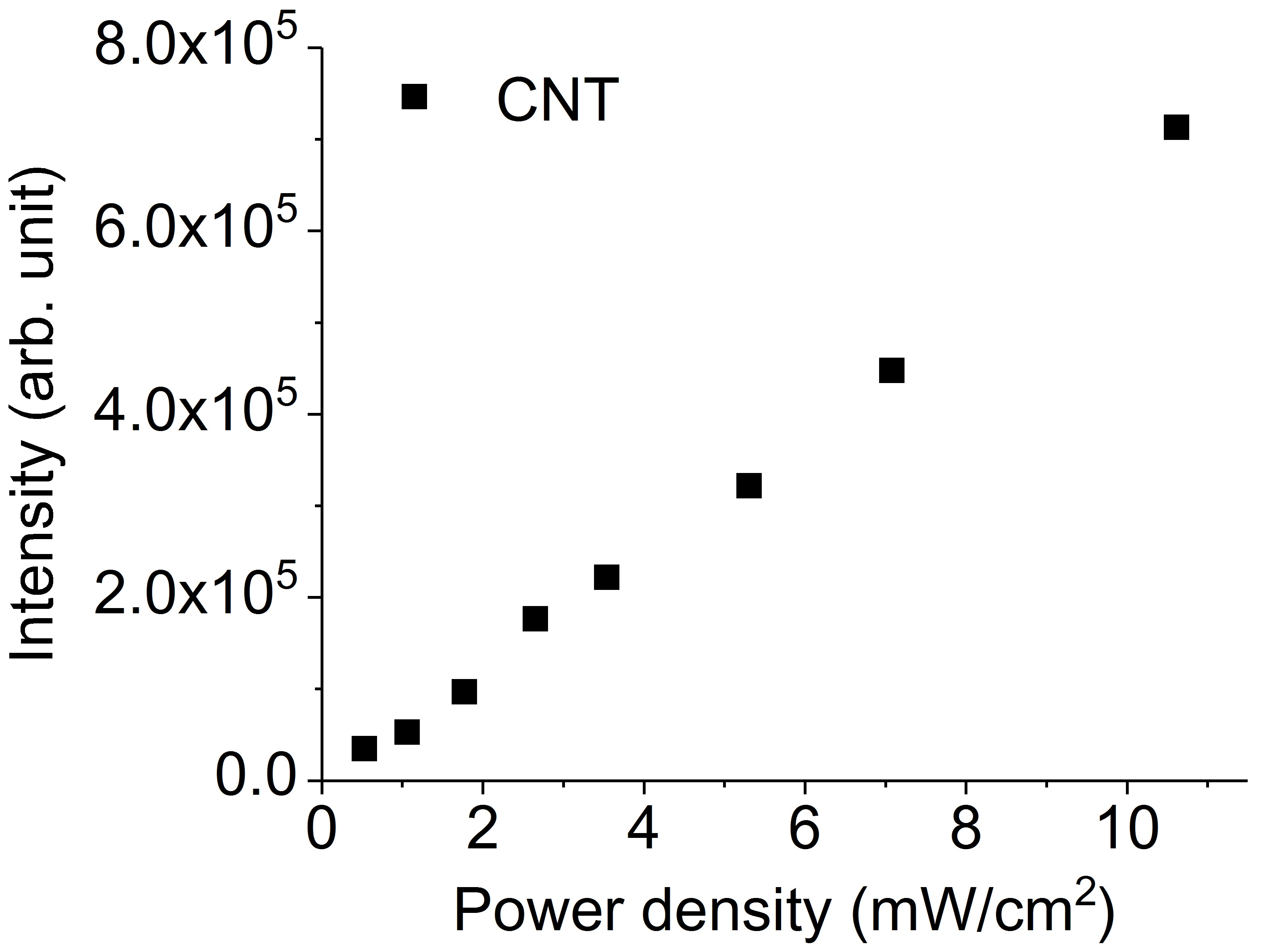


**Figure S2.** Luminescent intensities of the CNT dispersion sample measured using the developed V-trench biosensor with various power densities of the excitation light. The concentration of CNTs in the sample was 25 μg/mL. The excitation light was p-polarized. The intensity and power density are considered to be linear up to 11 mW/cm^2^. If the photothermal effect of CNTs affects the fluorescent signal, it would appear as non-linearity of the intensity against the power density. Therefore, for the excitation power below 11 mW/cm^2^, the photothermal effect would not affect the fluorescent signal of the developed sensor.
